# Human Plasmacytoid Dendritic Cells: From Molecules to Intercellular Communication Network

**DOI:** 10.3389/fimmu.2013.00372

**Published:** 2013-11-12

**Authors:** Till S. M. Mathan, Carl G. Figdor, Sonja I. Buschow

**Affiliations:** ^1^Department of Tumor Immunology, Nijmegen Centre for Molecular Life Sciences, Radboud University Nijmegen Medical Centre, Nijmegen, Netherlands

**Keywords:** cross talk, surface markers, T lymphocytes, viral infection, pDC migration

## Abstract

Plasmacytoid dendritic cells (pDCs) are a specific subset of naturally occurring dendritic cells, that secrete large amounts of Type I interferon and play an important role in the immune response against viral infection. Several studies have highlighted that they are also effective antigen presenting cells, making them an interesting target for immunotherapy against cancer. However, the modes of action of pDCs are not restricted to antigen presentation and IFN secretion alone. In this review we will highlight a selection of cell surface proteins expressed by human pDCs that may facilitate communication with other immune cells, and we will discuss the implications of these molecules for pDC-driven immune responses.

## Introduction

Within the heterogeneous dendritic cell (DC) family, two main subsets of naturally occurring blood DCs can be discriminated based on their phenotype and functional characteristics: myeloid DCs (mDCs) and plasmacytoid dendritic cells (pDCs). The mDC subset can be further divided in CD1c^+^ and CD141^+^, which show a high level of similarity in protein expression yet have also specific functions in the initiation of adaptive immune responses. CD1c^+^ mDCs have been shown to readily stimulate naïve CD4^+^ T cells and to secrete high amounts of IL-12 in response to toll-like receptor (TLR) ligation, whereas CD141^+^ DCs do not secrete much IL-12 but are well equipped to take up dead and necrotic cells for subsequent cross presentation of derived antigens to CD8^+^ T cells (1–4). In contrast to mDCs, pDCs have a very different protein expression profile reflecting their important and unique function in the secretion of IFN-α and anti-viral immune response (1, 2, 5). We and others have however recently demonstrated that like mDCs, pDCs are also very well capable of presenting both soluble and particulate exogenous antigens on both major histocompatibility complex (MHC) class I and II (6). In recent years, numerous studies have been performed to characterize the expression of pathogen recognition receptors (PRRs), TLRs, Fc receptors, C-type lectin (CTL) receptors, and other surface receptors on these cells (7–13). Furthermore, these studies have emphasized both similarities and differences between DC subtypes in their cytokine release profiles, and their ability to acquire, process, and present antigens (5, 14–17). These characteristics of the different DC subtypes have recently been reviewed extensively elsewhere (2, 4, 6, 18). Here, we will focus our attention specifically on pDCs, their role in immunity and, more specifically, their (potential) direct interactions with cells of the innate and adaptive immune system via cell surface molecules. Before going into detail about these cell surface receptors and how they mediate intercellular communication, we will first give a brief summary on general pDC function and localization to provide a context in which these intercellular communications take place. Although studies on murine pDCs are numerous, and commonalities between human and murine pDCs certainly exist, major differences between pDC of both species have also been reported. Therefore, in order to prevent confusion we limited ourselves to human pDCs unless explicit stated otherwise.

## pDC Function

A perturbation of the homeostatic condition that sets off the immune system can trigger either an immunogenic (immunostimulatory) or a tolerogenic (immunosuppressive) response, depending on the local circumstances and type of disease. By default, immature pDCs are tolerogenic, whereas activated (mature) pDCs can have both immunogenic and tolerogenic capacities depending on the local environment in which they are activated (19–21). pDCs are characterized as Lin^−^ MHC-II^+^ CD123 (IL3R)^+^ CD4^+^ CD303(BDCA-2)^+^ CD304(BDCA4; Neuropilin-1)^+^ and are mostly known for their ability to quickly produce large amounts of the Type I interferons (IFNs), IFN-α, and IFN-β, following viral infection, implicating pDCs as an important contributor during the early phase of anti-viral response (2, 22, 23).

The most important documented enveloped viruses known to stimulate Type I IFN release by pDCs are human immunodeficiency virus type 1 (HIV-1), herpes simplex virus (HSV), and influenza virus (24–27). Furthermore, parasites and bacteria containing DNA with unmethylated CpG sequences can trigger pDC activation (28–31). In addition to the anti-viral capacity, Type I IFN release by pDCs has also been reported to be important for pDC survival, (m)DC-mediated CD4^+^ and CD8^+^ T cell responses, mDC differentiation, cross presentation, upregulation of co-stimulatory MHC molecules and activation of natural killer (NK), and B cells (32–34).

Because of their expression of the endosomal TLRs TLR7 and 9, pDCs, in contrast to other (immune) cells, do not need to become infected to respond to viruses or intracellular bacteria (35, 36). TLR7 recognizes guanosine or uridine-rich, single-stranded RNA from viruses or synthetic products like guanosine analogs such as R848. TLR9 senses single stranded DNA containing unmethylated CpG motifs, which are usually found in bacterial and viral genomes, and additionally senses for synthetic oligonucleotides, such as CpG-ODN (37, 38). pDCs show differential responses based on the type of virus/bacteria that is recognized, which has been suggested to be attributed to a different site of TLR activation within the endosomal system (39). For example, depending on the subtype of CpG recognized (CpG-A, CpG-B, CpG-C) the outcome of the response can be different. While CpG-A, that triggers TLR9 in early endosomes, induces IFN-α release, CpG-B, signaling from late endosomes, leads to tumor necrosis factor α (TNF-α) and IL-6 production by pDCs (40). In addition, the interplay of the various PRRs tailors the pDC response to a specific pathogenic threat. In addition to TLRs, pDCs express several CLRs, including BDCA-2, DEC-205, dectin-1 and DCIR, and Fc receptor CD32, but they lack for instance DC-SIGN (22, 41–45). Although the full repertoire of receptors is still under investigation, most of these receptors drive antigen uptake, and in concert with TLR7 and 9, coordinate pDC-mediated immune responses.

## pDC Localization

Immature pDCs circulate in the blood but have been equipped with migratory capacities as they are found within lymph nodes (LNs), tumors, and near sites of viral/bacterial infection (46, 47). At all these sites pDCs are able to promote inflammatory responses by attracting other immune cells through chemokine release, and the subsequent modulation of these cells via cytokines or direct cell–cell interactions (48–51). However, in contrast to human myeloid mDCs or murine pDCs studies, reports addressing which inflammatory chemokines and adhesion receptors specifically drive migration of human pDCs are scarce (52). Human pDCs express chemotactic receptors C-C chemokine receptor type 7 (CCR7), chemokine (C-X-C motif) receptor 3 (CXCR3), CXCR4, and ChemR23 (CMKLR1) that likely mediate migration of pDCs into lymphoid organs and/or into inflamed tissue (48, 52–55). However, due to conflicting reports the role of classical lymphoid tissue CCR7^−^ Chemokine (C-C motif) ligand 21 (CCL21)/CCL19 pathways in resting human pDCs, is not conclusive yet (53, 56). Several studies show a high expression of CCR7 on “resting” blood DCs while others have reported a very low or a lack of expression on resting pDCs (53, 57–60). Similar to mDCs and murine pDCs, human pDCs upregulate expression of CCR7 upon TLR stimulation and migrate toward CCL21 molecules, suggesting an important role of CCR7 at least for the migration of mature pDCs to the LN (55). Furthermore IL-3 produced by T cells in the LN or by activated endothelial cells can lead to the upregulation of chemokine receptor 6 (CCR6) and CCR10 that may drive migration of activated IFN producing pDCs to inflamed skin or mucosa (61).

**Figure 1:**
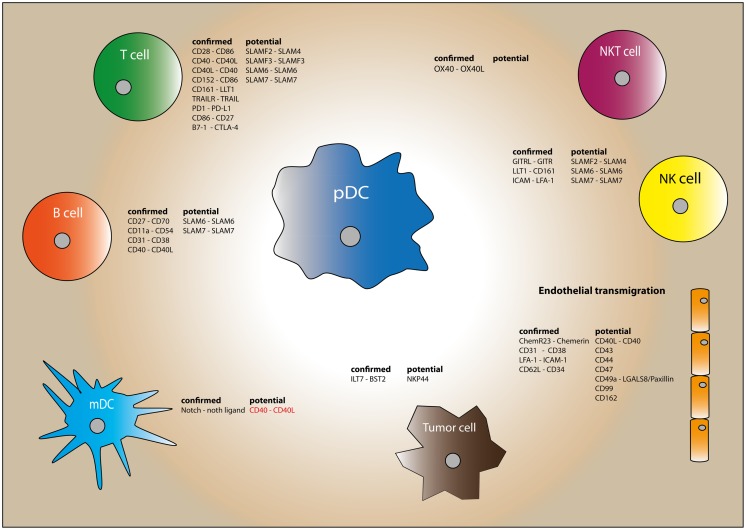
**Plasmacytoid dendritic cell have the capacity to interact with various immune cells through an array of surface molecules**. The expressed surface molecules of each cell type are divided into confirmed and potential interactions. The “confirmed” molecules have been reported to have a functional effect. Molecules listed in the “potential” column are molecules that have been found on human pDC but without functional data reported in literature. Molecules playing a potential role in humans, but already confirmed with functional studies in mouse are depicted in red.

In contrast to mDCs, which migrate from peripheral tissue to secondary lymphoid organs via afferent lymphatic vessels, pDCs have been described to migrate to the LN mostly directly from the blood via high endothelial venules (HEVs) (62, 63). Since pDCs first need to engage and traverse the endothelial cells lining of the blood vessels, endothelial cells likely represent the first cellular contact pDCs will engage in after leaving the blood stream. pDC would require a similar migration capacity to enter into inflamed or tumor tissue, which also requires interaction with endothelial cells and extravasation. Next, within the LN, or at the site of infectious or cancerous lesions, pDCs may encounter various immune cells. In the LN, pDC have been found in close contact with T lymphocytes, Invariant Natural Killer T (iNKT) cells, B lymphocytes, and NK cells (21, 24, 42, 64–66). At sites of infection pDCs might activate or get activated by mDCs and NK cells, whereas within the tumor microenvironment pDCs are known to interact predominantly with tumor cells and regulatory T (Treg) cells (67, 68). Below we have summarized the evidence reported thus far for each of these (potential) interactions, and the circumstances under which they occur (Figure 1).

## Endothelial Cells

**Figure 2:**
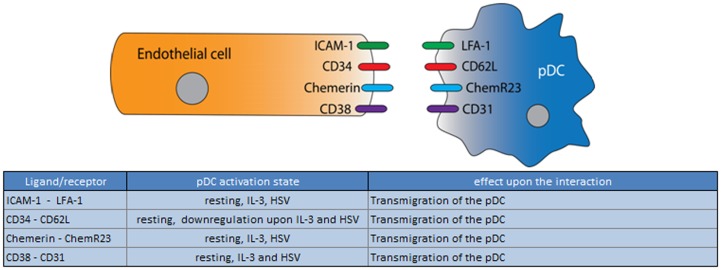
**Ligand/receptor paring of a pDC with an endothelial cell and the maturation state/activation stimuli associated with ligand or receptor expression on the pDC surface**.

Depending on their location (peripheral tissue or LN) and activation state, endothelial cells have been shown to express distinct cell surface molecules and to secrete a variety of chemokines and cytokines that may aid leukocyte transmigration and regulate the activation state of the migrating cells (69). Endothelial cells thus not only facilitate pDC transmigration into the site of infection, the tumor lesion, or the LN but may also have the potency to influence pDCs mediated immune responses trough pro- or anti-inflammatory cytokines as well as growth factors (69). Indeed, endothelial cells also produce IL-3 and VEGF that bind and trigger pDC marker proteins CD123 and BDCA4 respectively, and likely will promote pDC survival and migration after crossing the endothelial barrier. Documentation however of the crosstalk between human pDCs and endothelia is scarce and limited to a few recent studies that we will discuss. Intriguingly, and in contrast to murine pDCs, both resting and matured human pDCs (stimulated by influenza virus) uniquely express the receptor for chemerin, ChemR23 (48). Chemerin is present on the surface of endothelial cells in the lumen of HEVs as well as in blood vessels of inflamed tissue. The interaction between endothelial cell-bound chemerin and pDC ChemR23 seems to play a crucial role in the migration of pDC from the blood both into LNs and into inflamed tissue (Figure 2) (48, 70, 71). Like pDCs, T cells also migrate from the blood to the LN via HEVs and thus pDCs may exploit a similar set of molecules as used by T cells. Indeed, pDCs express adhesion molecules CD31, CD43, CD44, CD47, CD62L, CD99, and CD162 (SELPLG, CLA) that may play an important role in the tethering and rolling of pDCs on endothelial cells, but for most of these molecules, functional data for a role on human pDCs is lacking (54, 72, 73). The Lymphocyte function-associated antigen 1 (LFA-1) and very late antigen 1 (VLA-1) (CD49a/CD29) molecules might play an important role in subsequent firm adhesion and transmigration of pDCs (72). Although the expression of all these molecules was initially only detected by microarray, with the exception of CD44, most were confirmed by flow cytometry (74). Furthermore, flow cytometry demonstrated that expression of both CD62L (moderately) and CD99 was downregulated upon exposure to IL-3 and HSV, indicating that activated pDCs may take different migratory routes compared to their immature counterparts (74). While immature pDCs express CD62L and use HEVs to migrate into the LN, downregulation of CD62L on mature pDCs suggests that these cells enter LN without passing HEV, but rather through the lymphatic vessels. Furthermore, another study identified a cleavage of CD62L after entering the HEV suggesting this molecule may have become obsolete for pDCs following this pathway (75). In skin, in contrast, after transversing the vessel wall expression of CD62L on pDC remains high, indicating that in this case it may still have a function at a later stage (54).

In summary, although there is evidence for Intercellular Adhesion Molecule 1 (ICAM-1)/LFA, CD31/CD38, and CD34/CD62L interaction between pDCs and endothelial cells, until now, only the chemerin/ChemR23 interaction has been conclusively demonstrated to play a role during the migration process. The migratory function of the other adhesion molecules reportedly expressed by human pDC are currently only hypothetical and based on knowledge from other leukocytes or murine cells. Furthermore also the role of endothelia in the regulation of the pDC activation state awaits further study.

## T Cells

**Figure 3:**
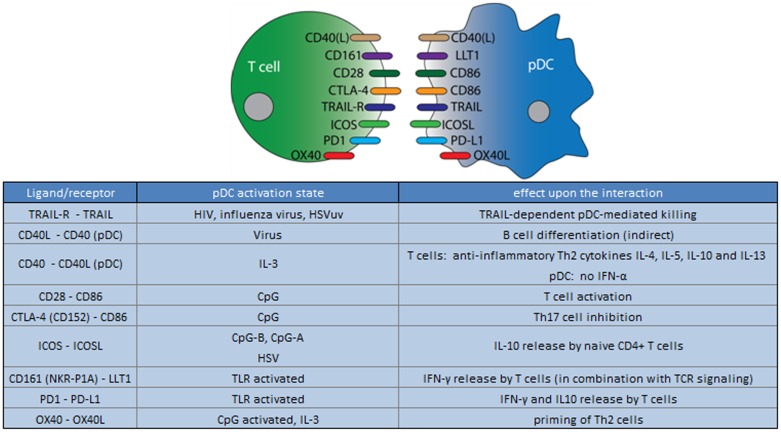
**Ligand/receptor paring of a pDC with a T cell and the maturation state/activation stimuli associated with ligand or receptor expression on the pDC surface**.

During infection, immature DCs located in the inflamed tissue get activated through pathogenic interaction and pro-inflammatory cytokines. Mature (activated) DCs subsequently translocate to the LN and induce naïve T cells to differentiate into effector T cells. Based on the repertoire of danger signals, effector T cells will have different characteristics and will evoke a different immune response. pDCs have an important role in coordinating such an immune response, since the molecules involved in the interaction between DCs and T cells determine T cell polarization (Th1, Th2, Th17). Numerous studies have established that pDCs are *bona fide* antigen presenting cells (APCs), capable of presenting exogenous antigens on both MHC class I and II molecules and thus can trigger both CD4^+^ T helper (Th) cells and CD8^+^ cytotoxic T cells (5, 26, 76–78). The nuances of pDCs antigen processing and presentation have recently been reviewed by Guery and Hugues (42) and Nierkens et al. (79). Here, we focus our attention on how pDC cell surface receptors may skew T cell function (Figure 3). Freshly isolated (immature) pDCs are known to induce CD4^+^ T cell anergy presumably because they lack co-stimulatory molecules; conversely, activated pDC clearly induce a broad spectrum of T cell differentiation, for example, Th1, Th2, Th17, and Treg, based on the cytokines secreted and cell surface proteins expressed (21, 80–84). Like mDCs, activated pDC express high levels of MHC molecules and the co-stimulatory molecules CD80 (B7-1), CD86 (B7-2), and CD83 to present antigens and fully license and activate T cells (5, 6). Several studies have demonstrated that (virally) matured pDCs, through the release of cytokines, mostly induce a Th1 phenotype (IFN-γ/Il-12 in response to CpG, virus) but Th2 (IL-4) and Th17 (IL-17) skewing has also been reported when pDC are activated with IL-3 or CD40 and TLR7 ligands, respectively (82, 85–87). Furthermore IL-21 (produced in the LN) was shown to trigger the release of Granzyme B by TLR-activated pDCs thereby dampening CD4^+^ T cell proliferation (88). Together these studies show how pDCs may regulate immune responses. Apart from cytokines released by pDCs, several pDC surface receptors may directly affect T cell skewing and function, including the inducible T-cell co-stimulator ligand (ICOSL). pDCs express ICOSLG when activated by CpG-(A, B, and C) IL-3/CD40L or virus (Flu/HSV) (83). ICOSLG is the ligand for the T-cell-specific cell surface receptor inducible costimulator (ICOS) and has been shown to trigger naive CD4^+^ T cells to produce IL-10 during both pDC Th1 or Th2 skewing in response to CpG/virally or IL-3/CD40L-matured pDCs, respectively (83, 84). It has been suggested that ICOSL-activated pDCs generate IL-10 producing Tregs to dampen immune responses, preventing excessive inflammation (83). Furthermore TLR activated, but not resting pDCs and mDCs, express programed death receptor-ligand 1 (PD-L1), which may induce T cells anergy/suppresses T cell activation by binding to its receptor, program death ligand 1 (PD1), which is expressed by T cells (89, 90). The immunosuppressive effect of PD-L1 has been confirmed by using blocking antibodies on DCs, and additionally in follow-up studies where blocking the PD-L1/PD1 interaction lead to “enhanced tumor-specific T cell expansion and activation” (6, 91, 92). The surface receptor OX40, which is expressed on IL-3 activated pDCs, can induce a Th2 T cell response resulting in IL-4, IL-5, and IL-13 release by CD4^+^ T cells (93, 94).

Furthermore, after stimulation either with synthetic TLR7 and 9 agonists or with the natural TLR7 agonists, like influenza virus or UV-inactivated HSV type 1(HSV_UV_) pDCs can induce programed cell death/apoptosis, by expressing tumor necrosis factor-related apoptosis-inducing ligand (TRAIL) (74, 95, 96). TRAIL expression on pDCs uniquely correlates with viral load, and the capacity to kill HIV-infected CD4^+^ T cells by binding to the TRAIL receptor, a process described as “TRAIL-dependent pDC-mediated killing” (97). However, given the very limited cell numbers, it remains to be seen how important TRAIL^+^ pDCs are in clearing a viral infection via the direct killing of infected cells (97, 98).

Another surface molecule expressed on TLR-activated pDCs that may affect T cell function is the lectin-like transcript 1 (LLT1), which in addition to activated pDCs, is expressed by most activated lymphocytes (including B cells, T cells, and NK cells) and mature monocyte-derived DCs (99). LLT1 is a ligand of CD161 (NKR-P1A), which is expressed by subsets of T cells (e.g., Th1, Th17, and a subpopulation of CD8^+^ T cells) and NK cells. When ligated LLT1 triggers T cell proliferation and IFN-γ secretion as well as inhibition of NK cell cytotoxicity (99–102). Thus, LLT1 on pDCs may serve as a co-stimulatory molecule, and after binding to CD161 expressing T cells, could drive proliferation and IFN-γ secretion (51).

So far, we discussed how pDC receptors may affect T cell function but of course, conversely, T cells may also influence pDC function. In a multicellular immune cell signaling cascade the presentation of viral antigens by pDCs brings about IL-2 release by T cells as well as CD40L expression. T cell CD40L upon binding to CD40 on pDCs, triggers IL-6 release by pDC, which in turn enables B cell plasma blasts to become antibody-secreting plasma cells (Figure 8) (21, 64).

In summary, while immature pDCs predominantly induce T cell anergy, their activated counterparts may have either inhibitory or activating effects on T cells. Which of the latter in the case depends on stimuli that trigger pDC maturation and which cytokines and surface molecules are expressed as a result. Thus pDCs play pivotal role in T cell activation and fine tuning of the adaptive immune response.

## iNKT Cells

Natural Killer T (NKT) cells form a specialized T cell subset expressing a semi-invariant T cell receptor (TCR-αβ) and surface antigens traditionally associated with NK cells. The unique TCR on their cell surface enables NKT cells to recognize glycolipid antigens rather than peptides, presented in the context of the MHC class I-like molecule, CD1d (103). The most well characterized subset of NKT cells are called iNKT cells, since they express an invariant TCR-α chain, and are reactive to the potent NKT cell agonists α-galactosylceramide (α-GalCer) (103).

**Figure 4:**
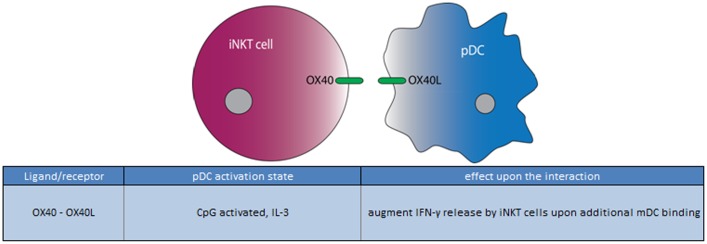
**Ligand/receptor paring of a pDC with an iNKT cell and the maturation state/activation stimuli associated with ligand or receptor expression on the pDC surface**.

Studies have shown that pDCs interact with iNKT cells directly, both via cell–cell interactions and by cytokine release (104). In contrast to the mDCs, pDCs lack the expression of CD1d, which is an important molecule for crosstalk with iNKT cells (105). Nonetheless, over the past few years the ability of iNKT cell to “sense” subtle changes within their microenvironment in a CD1d-independent mechanism, uncovered that cytokines released by pDCs are essential (106, 107). Indeed, CpG activated pDCs upregulate activation markers on iNKT cells via TNF-α and IFN-α release, and selectively enhance double-negative iNKT cell survival but not that of other NKT cell populations (104). However, the interplay of iNKT cells with pDCs alone is not sufficient for iNKT expansion and does not lead to a cytokine release by iNKT cells. Rather, the CpG activated pDCs enables the iNKT cells to productively interact with CD1d expressing mDCs, thus initiating an immune response (61). Both iNKT cells and mDCs lack expression of TLR9 and are therefore unresponsive to CpG; hence, cytokines released upon ligation of TLR9 on pDCs modulate the tissue microenvironment. Not only cytokines, but also a direct interaction between pDCs and iNKT cells may be of importance; CpG-stimulated pDCs express the ligand CD252 (OX40L), which binds CD134 (OX40) present on the surface of iNKT cells, and augments IFN-γ release by iNKT cells in response to lipid antigen presentation by mDCs (Figure 4) (66). Further support for such a direct interaction between pDCs and iNKT cells via OX40L/OX40 comes from murine studies (108–110).

In summary, the interaction between iNKT cells and pDCs can be both via cytokines and via direct cell interaction. The role so far seems to facilitate the activation of iNKT by CD1d expressing mDCs. This may become important particularly in situation when TLR9 ligands are available. So far, only OX40L/OX40 are known to play a role in the direct pDC/iNKT cell cross talk.

## B Cells

**Figure 5:**
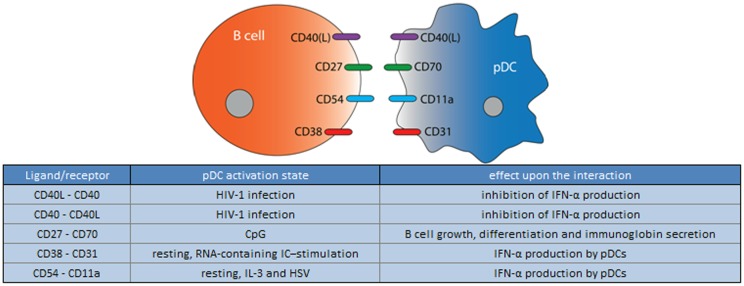
**Ligand/receptor paring of a pDC with a B cell and the maturation state/activation stimuli associated with ligand or receptor expression on the pDC surface**.

B cells are the only cells that produce antibodies, and therefore, have a critical role in the humoral immune response. Release of Type I IFNs by pDCs leads to an increase of TLR7 and several activation markers on B cells (111, 112). Moreover, as outlined above, pDCs, in concert with T cells, control B cell differentiation into plasma cells via the secretion of IFN-α and IL-6 (64). In addition, pDCs can affect B cells via direct cell–cell contact. Several studies have shown the importance of CD40-CD40L interactions between B cells and pDCs (Figure 5) (24, 64, 65). In addition, upon activation with CpG, pDCs were demonstrated to interact with B cells via CD70/CD27 molecules. This interaction results in B cell growth, differentiation, and immunoglobulin secretion (113).

Furthermore, recent *in vitro* studies have shown that activated B cells are able to stimulate matured pDC to produce IFN-α by direct cell–cell contact (114). Blocking the surface molecules OX40L, CD27, CD40, or CD40L with monoclonal antibodies did not influence the effect of B cells on pDC-derived IFN-α production. However, the IFN-α production by pDCs was significantly reduced when blocking LFA-1 or PECAM-1 (CD31) by 50% and 80%, respectively, indicating that these molecules are at least partially responsible for B cell mediated pDCs activation (114).

Taken together, pDCs and B cells are able to induce reciprocal cytokine release and activation by both soluble mediators and direct cell–cell interaction, and so far have been found to be predominantly stimulatory in nature (64, 113, 114).

## mDCs

Synergism of mDCs and pDCs are not restricted to the activation of NKT cells. pDCs and mDCs have been demonstrated to be in close contact *in vivo* at steady state as well as under inflammatory conditions, and it has been suggested that they act synergistically to induce more potent immune responses (115–117). Upon stimulation both mDCs and pDCs function as APCs and follow a similar maturation program, and express the co-stimulatory markers CD40, CD80, CD83, and CD86 to interact with T cells (17, 118). However, there are complementary differences especially in the expression of PRRs (e.g., TLRs, CLRs) and thus in their response to pathogenic triggers. Whereas mDC subtypes express TLR1, 2, 3, 4, 5, 6, 8, and 10, but no TLR7 and 9, the expression of these TLRs on pDCs is the exactly opposite except from TLR2 and 10, which are shared (7, 8, 35, 119–122). pDCs respond to TLR7 and 9 ligands with large amounts of IFN-α and TNF-α (123). In contrast, mDCs release very different cytokines, primarily IL-1β, IL-8, IL-6, IL-10, IL-12p70, TNF-α to variable extents, upon triggering of their TLRs (7, 118, 122). Upon viral infection pDCs are known to respond quicker and with larger amounts of cytokines than mDCs (124). Thus pDCs and mDCs have non-overlapping sensitivities to invading pathogens, and accumulating reports suggest that pDC and CD1c-mDC may cross-activate each other for a more effective immune response. Crosstalk may occur in a paracrine fashion through cytokines like Type I IFNs and TNF-α but also via direct cell contact (118, 125). In a paracrine fashion TNF-α expressed by pDC can cross-activate co-cultured CD1c-mDCs (126). However, there is clear evidence to suggest that CD1c-mDCs and pDCs in other cases require close contact for some parts of this crosstalk, until now it is unclear what molecules are involved (118). Recent studies by Piccioli et al. implies that several members of the TNF family, CD40L/CD40, OX40L, HEVML, RANKL, CD27, CD30L, glucocorticoid-induced tumor necrosis factor receptor-ligand (GITRL), and 4-1BB are redundant in the CD1c-mDC/pDC cross talk (9). Experimental evidence for the absence of a role for any of these interactions however was so far not reported but only came from unpublished blocking experiments mentioned in these studies, making it extremely hard to deduce whether these interactions can and should be excluded completely (9, 118). Interestingly, murine models do suggest that the TNF member CD40/CD40L may have a crucial role in the CD1c-mDC/pDC cross talk, yet this result needs to be recapitulated in human CD1c-mDC/pDC assays (9).

**Figure 6:**
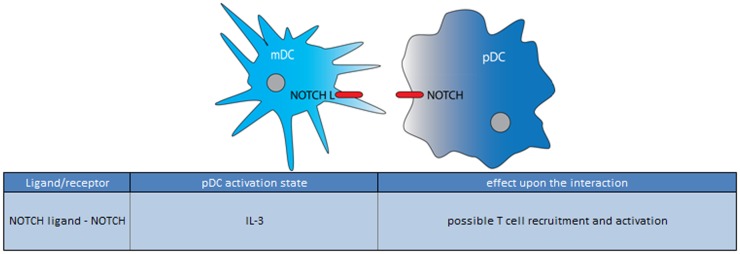
**Ligand/receptor paring of a pDC with a mDC and the maturation state/activation stimuli associated with ligand or receptor expression on the pDC surface**.

So far only for the NOTCH receptor-ligand interaction evidence is provided for a role in the communication between pDCs and CD1c-mDCs but again experimental evidence is scant (Figure 6) (117). With co-culture experiments they demonstrated that LPS-activated CD1c-mDCs caused an upregulation of maturation marker (CD25, CD86) on the pDC surface and increased IL-6 and CCL19 release in the supernatant. To confirm the involvement of NOTCH pathway, experiments with γ-secretase/NOTCH inhibitor DAPT and soluble NOTCH ligands were preformed and showed a reduced effect on Notch target genes. Activation of the NOTCH pathway upon CD1c-mDC/pDC interaction suggests that this intercellular contact promotes an immune stimulatory response, however, further experiments are needed to unravel the exact mechanism and other molecules potentially involved in this CD1c-mDC/pDC cross talk (117).

Another possible candidate for the interaction between pDC and CD1c-mDC is ICAM-1, expressed on both, pDCs and mDCs, and known as an widespread adhesion molecule with co-stimulatory activity on other immune cells (127). ICAM-1 was found to be strongly upregulated on pDC upon stimulation with TLR9 ligand CpG, while its matching receptor LFA-1 (CD11a/CD18) is constitutively expressed on CD1c-mDCs (9).

Taken together, there is clear evidence that direct cellular interactions are indeed important for CD1c-mDC/pDC cross talk in humans, similar to what was observed in murine studies. However, besides NOTCH receptor-ligand interactions, any experimental evidence that argues in favor of or against the involvement of other specific receptor-ligand interactions is so far lacking (9, 118).

## NK Cells

**Figure 7:**
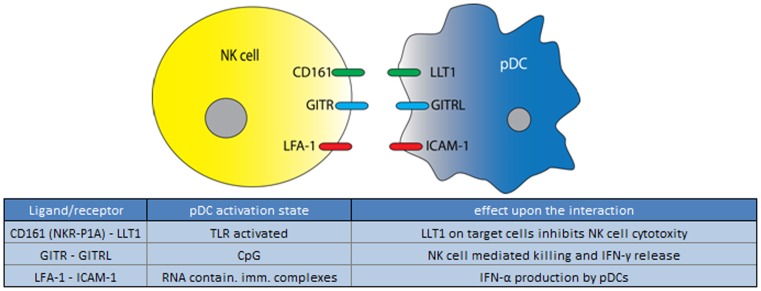
**Ligand/receptor paring of a pDC with an NK cell and the maturation state/activation stimuli associated with ligand or receptor expression on the pDC surface**.

Natural killer cells belong to the innate immune system and are able to respond rapidly to virally infected cells and to tumor formation. This is due to their unique ability to recognize stressed cells or the absence of MHC on the surface of infected or malignantly transformed cells, and their subsequent ability to lyse these cells. The bi-directional pDC-NK cell interaction is known to play an important role in host defense, and again is mediated both by cytokines and via direct cell contact (128, 129). Type I IFNs secreted by pDCs have long been known to enhance the cytolytic potential of NK cells, and NK cells co-cultured with pDCs are more activated, and have increased cytolytic activity (49, 50, 130–133). pDCs and NK cells have been found in close proximity in the T cells areas of human tonsils (50). In addition, during infection or in case of a malignancy, pDCs and NK cells may migrate simultaneously to the site of the lesion, for example during Herpes simplex infection (25). These reports demonstrate the ample opportunities for these cells to engage in direct interactions, which is further supported by the findings that, when co-cultured, pDCs and NKs cells readily interact (134). Upon stimulation by a virus or CpG, pDCs express GITRL that can bind GITR expressed by NK cells (Figure 7). Via the (GITRL)-GITR interaction mature pDCs enhance NK cell mediated killing as well as IFN-γ production. To affect NK cells, however, pDCs expressing GITRL do require the simultaneous presence of IFN-α (50). Furthermore, while the upregulation of CD69 on the surface of NK cells depends on the release of IFN-α and TNF-α by mature pDCs, upregulation of HLA-DR on the surface of a subpopulation of NK cells depends on direct pDC-NK cells contact (49). HLA-DR expressed on NK cells is thought to play an important role in handling bacterial infections such as *Mycobacterium bovis* (BCG) (135). Although the interaction responsible for HLA-DR upregulation remains to be elucidated, it is known that the maturation state of the pDC is not important for the induction of HLA-DR expression on NK cells, indicating the HLA-DR inducing factor is not affected by pDC maturation (49, 130, 132, 136).

The bi-directional crosstalk between pDCs and NK cells also affects pDC function; IL-2, immune complex or IL-12/IL18-stimulated NK cells induce pDCs to release IFN-α which was shown to depend largely on LFA-1-mediated interactions between NK cells and pDCs, and to a lesser extent on NK cell secreted MIP1α (132, 134). LFA-1 and FcγRIIIA on the pDC also increase cytokine release by NK cells (137). Furthermore, IL-2 stimulated NK cells induced pDCs to express the maturation marker CD83, but not CD80 and CD86, in a contact dependent manner, which also indicates the existence of different stimulatory pathways that can induce expression of different maturation markers on pDCs (132).

Contact with NK cells potentially puts pDCs in danger of becoming lysed. However, immature pDCs are protected from NK cell mediated lysis, and this is at least partly due to the high expression of HLA class I, and the absence of Nectin-2, the ligand for NK cell activating receptors DNAM-1 (132, 138). Culture of pDCs with IL-3 however causes the upregulation of Nectin-2 on pDCs, and makes them more susceptible to DNAM-1 and NKp30-mediated killing (138). Activation of pDCs by TLR7 and 9 may help to prevent NK cells lysis as they express the LLT1 (LLT1 or CLEC2D; above), which is a ligand of NK cell surface protein P1A (NKR-P1A; CD161). P1A is expressed by both NK and NKT cells and when ligated inhibits NK cell cytolytic function and IFN-γ release (51, 99, 101, 139). Taken together, pDCs in various modes of action, seem to be differentially susceptible to NK cell-mediated lysis through the absence of activating NK cell receptor-ligands, as well as the regulated expression of ligands for NK cell inhibitory receptors. Also, high MHC I expression is protective.

**Figure 8:**
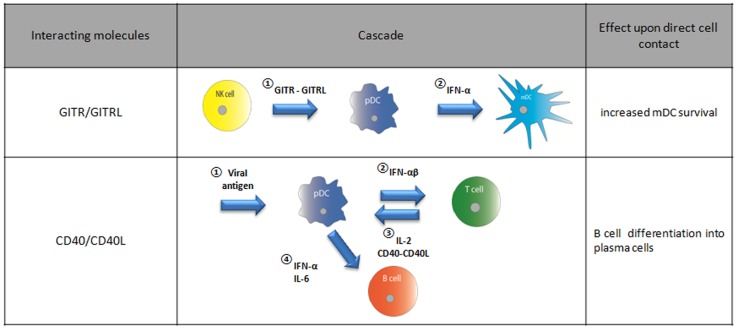
**Direct cell interaction dependent on GITR/GITRL or CD40/CD40L binding and their effect on a certain cell type**.

In summary, non-lethal pDC/NK cell interactions seem to play an important role in enhancing the early immune response to a viral or bacterial infection as pDCs activate the NK cell by producing IFN-α and via GITRL. This feed-forward system likely promotes NK cells to rapidly lyse infected cells (132). NK cell activity in turn induces a further increase of IFN-α by pDCs and promotes their maturation, which may in turn increase the recruitment and survival of mDCs (Figure 8).

## Tumor Cells

Several early studies have reported decreased numbers of pDCs and mDCs in the blood of patients suffering from various types of cancers (140–143). However, a recent study with melanoma patients detected no significant difference between the levels of immature pDCs in healthy donors and patients (144). Compared to healthy volunteers, pDCs derived from melanoma patients did, however, show a higher expression of CCR6, and increased ability to migrate toward Chemokine (C-C motif) ligand 20 (CCL20), a ligand for CCR6 (144). CCL20 is expressed by keratinocytes in the skin and by melanoma cells, suggesting that the CCL20/CCR6 interaction is involved in the pDC migration process from the blood to the tumor (145–147). Indeed, high pDC infiltration have been observed in many types of cancer including melanoma, head and neck cancer, ovarian and prostate cancer, and these infiltrates mostly negatively correlate with patient survival. On the other hand, an increase of pDCs in tumor-draining LN may be beneficial [reviewed in (143, 148)]. pDCs infiltrated in tumor microenvironment are mainly immature, and therefore seem to be predominantly immunosuppressive/tolerogenic (148). In recent years, evidence has accumulated that tumors may block anti-tumor response by maintaining pDCs in an inactive/tolerogenic state. Mechanisms responsible for keeping the pDC in this state include the secretion of prostaglandin 2 (PGE_2_) and TGF-β, which, in a synergistic manner inhibit pDC-derived IFN-α and TNF-α production in response to TLR7 and 9 ligands, as well as inhibiting CCR7 expression, thereby impairing the migration of pDCs to the tumor-draining LN to prime T cells with tumor antigens (148–150). In addition, there is evidence that PGE_2_-stimulated pDCs indirectly support tumor cell proliferation, migration, and invasion, as well as tumor angiogenesis, via the release of IL-6 and IL-8 (151–158). Furthermore, tumor-resident pDCs may also influence tumor growth indirectly through the induction of Tregs. In epithelial ovarian cancer the majority of Foxp3^+^ Treg cells accumulating in the tumor microenvironment expressed the ICOS, and tumor pDCs expressing the ICOSL were shown to be essential for the expansion and suppressive function of these regulatory Foxp3^+^ Tregs (67, 68).

**Figure 9:**
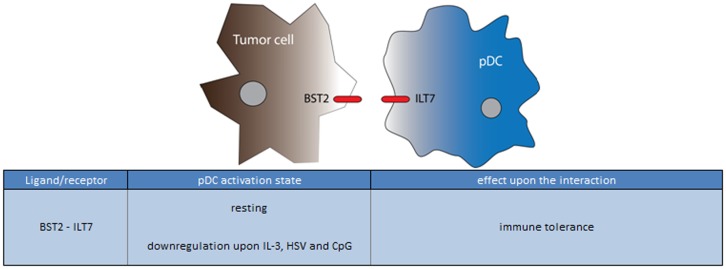
**Ligand/receptor paring of a pDC with a tumor cell and the maturation state/activation stimuli associated with ligand or receptor expression on the pDC surface**.

On their surface, unstimulated pDCs (uniquely with respect to all other leukocytes) express the immunoglobulin-like transcript 7 (ILT7) protein, which is activated by binding to bone marrow stromal cell antigen 2 (BST2, CD317; reviewed in (159). BST2 is expressed on human cancer cells, monocytes, and vascular endothelium in response to IFN-α (Figure 9) (160–162). Similar to BDCA-2, ILT7 forms a complex with FcεRIγ, which, when ligated activates an immunoreceptor tyrosine-based activation motif (ITAM)-mediated signaling pathway that dampens TLR-7 and 9-induced IFN-α and TNF-α production (163, 164). ILT7 is downregulated upon stimulation of the pDCs by CpG, HSV, or IL-3, suggesting that pDC maturation prior to entering the tumor site may partly protect it from this suppressive mechanism (159, 164, 165). In addition to ILT7, immature pDCs also express the ITIM motif containing receptors ILT2 and ILT3 that bind to MHC class I molecules, and an unknown ligand, respectively. Both receptors are associated with immune tolerance, probably through the suppression of T cell responses, and in agreement with this notion, these receptors are downregulated upon pDC activation (166–168). Whether these molecules may also have an active role in the pDC-tumor cell interaction, or rather regulate pDC-T cell activation, remains to be investigated. Likewise, there may be a yet unappreciated role for pDC expression of NKp44, which has been demonstrated to down modulate pDC IFN-α responses upon ligation, and may be utilized by the tumor to dampen pDC-mediated immune responses (169). Indeed, an inhibitory NKp44 ligand complex containing HLA I and PCNA was recently reported to be expressed by several tumor cells (159, 165, 170).

So far, most studies point out an immune suppressive role for pDCs favoring tumor progression, however several other studies demonstrate that the situation may be very different if pDCs are properly activated. In this case pDCs may trigger anti-tumor T cell-mediated immune responses (above) or even actively kill tumor cells (171, 172). As previously discussed, in relation to the T cell/pDC interaction, activated pDCs express TRAIL, which induces an apoptotic process by binding to the TRAIL receptors. Tumor cells are known to be sensitive to TRAIL, and via this interaction could directly induce tumor cell apoptosis (171). Avoiding this apoptotic pathway by downregulation of TRAIL has been reported for several cancers by numerous studies (173).

Taken together, the suppressive tumor microenvironment decreases the immune stimulatory functions of pDCs resulting in tumor progression. Preventing these processes, while at the same time activating pDCs, forms a promising target for anti-tumor therapies. Moreover, in a recent clinical feasibility study in our department, we demonstrated that the administration of autologous *ex vivo*-matured tumor antigen loaded pDCs proved to be successful and induced objective clinical responses in several patients (60).

## Less Explored in pDC Cellular Interactions: The SLAM Family

Above we have provided an overview of the interactions pDCs likely engage in during their lifecycle, as based on experimental evidence and *in vivo* proximity. In addition we discussed the molecules likely to participate in these interactions. There is, however, a poorly understood family of proteins which are highly expressed pDCs, and which deserve more attention. This is the SLAM family of receptors, for which a role in a diverse range of cellular interactions is highly suspected, yet currently unexplored. Five family members are expressed on the pDC surface (largely) independent of its activation state: SLAMF2 (CD48; BLAST1), SLAMF5 (CD84), SLAMF3 (CD229; Ly9), SLAM7 (CD319, CRACC), and NTBA (CD352) (74, 102). Except for SLAMF2 (below), these proteins have in common that they engage mostly in homotypic interactions; such interactions may occur between homotypic cells, but also between different cell types, opening up the possibility that these molecules mediated direct cell interactions of pDCs with each other, or with others cells also expressing these receptors (174). Homotypic SLAM family interactions have the ability to regulate cell activation and proliferation as well as cytolytic activity (174). SLAMF5 is highly expressed by many immune cells, and has been shown to play a role in T cell-B cell adhesion, and for optimal germinal center formation (175, 176). Furthermore SLAMF5 was detected on leukemic pDCs, and can work as an inhibitor for FcεRI-mediated signaling in mast cells (177, 178). SLAMF3 is also expressed on T cells. Here it reduces IFN-γ production and ERK activation upon stimulation, and thus via this molecule pDCs might trigger a similar response (175, 179). SLAM7, in contrast, is widely expressed on activated B lymphocytes, NK cells, and CD8^+^ T lymphocytes (74, 180–182) and has been shown to promote B cell proliferation, activate NK cell cytotoxicity (but not NK cell proliferation) (180, 182, 183). Finally, NTBA in addition to pDCs, is present on NK, T, and B cells where it may affect cytotoxicity as well as the IFN-γ and TNF-α release (174, 184, 185). Interactions between NTBA on pDCs and NK cells may therefore have the potential to positively regulate both NK cells and pDCs.

In contrast to the other family members, SLAMF2 which is also present on the surface of pDCs, engages in a heterotypic interaction with family member 2B4 (CD244), and could thus play a role in the interaction of pDCs with 2B4-expressing NK or T cells (74, 175, 186). SLAMF2 via 2B4 can activate NK cells (186).

Overall, although experiments are largely lacking, the presence of such a high number of SLAM family members on pDCs, their homotypic interactions, as well as the known effects of their triggering on other immune cells makes us speculate that pDCs may very well exploit these receptors to communicate with other immune cells.

## Conclusion and Outlook

In this review we summarized the existing data which supports the idea that during their life cycle, human pDCs interact with numerous immune cells. Additionally, we have attempted to provide a contemporary overview of the molecules that drive these interactions, and the consequences of their expression on pDCs. It is clear that pDCs play a pivotal role in ensuring a rapid immune response, especially upon viral infection, by strong IFN-α release, but also via direct cell–cell interaction. Depending on the pDC activation state, cytokines released by pDCs and direct pDCs surface receptors may inhibit or activate other immune cells. This large influential capacity of pDCs suggest that they are master regulators of both innate and adaptive immune responses. Besides secretion of the highly potent yet broadly acting IFN-α, pDCs have a highly versatile repertoire of cell surface molecules to further fine tune immune responses. These characteristics make them an interesting and potential highly valuable therapeutic target to be exploited or targeted in cancer therapy, infectious or autoimmune disease. Importantly, controlling cytokine secretion and the surface expression of specific receptors is essential to steer the immune response into the desired direction. In cancer therapy, lifting the suppressive actions of tumor-resident pDCs may greatly enhance existing/endogenous anti-tumor immune response (187). In addition, anti-cancer immune responses may be initiated or boosted by vaccination with autologous tumor antigen loaded pDCs (60). A preliminary clinical trial using pDCs in melanoma vaccination therapy, carried out by our department, has demonstrated the use of thick born virus vaccine (FSME)-matured pDCS in cancer vaccination is safe and feasible, and despite low patients numbers, showed an improved survival of pDCs vaccinated patients (60). In particular, the extremely low doses ranging from 0.5 to 3 million pDC per patient demonstrate the potency of these cells. The exact reason for the success of pDCs however is not yet completely understood, but in addition to IFN-α production and the induction of tumor-specific T cells, we envision there may also be a significant role for the effective combination of surface receptors expressed by the pDCs and their resulting interaction with other immune cells.

In conclusion, research over the past few years has greatly increased our knowledge of the repertoire of pDC-expressed surface receptors and cellular interaction partners, and has emphasized that there is more to the pDCs than IFN-α alone. Importantly, however, further studies are required to identify the role of these molecules and interactions in pDC function and immune responses in general.

## Conflict of Interest Statement

The authors declare that the research was conducted in the absence of any commercial or financial relationships that could be construed as a potential conflict of interest.
